# Compression of next-generation sequencing quality scores using memetic algorithm

**DOI:** 10.1186/1471-2105-15-S15-S10

**Published:** 2014-12-03

**Authors:** Jiarui Zhou, Zhen Ji, Zexuan Zhu, Shan He

**Affiliations:** 1 College of Biomedical Engineering and Instrument Science, Zhejiang University, Hangzhou, 310027, China; 2 Shenzhen City Key Laboratory of Embedded System Design, College of Computer Science and Software Engineering, Shenzhen University, Shenzhen, 518060, China; 3 School of Computer Science, University of Birmingham, Birmingham, B15 2TT, UK

## Abstract

**Background:**

The exponential growth of next-generation sequencing (NGS) derived DNA data poses great challenges to data storage and transmission. Although many compression algorithms have been proposed for DNA reads in NGS data, few methods are designed specifically to handle the quality scores.

**Results:**

In this paper we present a memetic algorithm (MA) based NGS quality score data compressor, namely MMQSC. The algorithm extracts raw quality score sequences from FASTQ formatted files, and designs compression codebook using MA based multimodal optimization. The input data is then compressed in a substitutional manner. Experimental results on five representative NGS data sets show that MMQSC obtains higher compression ratio than the other state-of-the-art methods. Particularly, MMQSC is a lossless reference-free compression algorithm, yet obtains an average compression ratio of 22.82% on the experimental data sets.

**Conclusions:**

The proposed MMQSC compresses NGS quality score data effectively. It can be utilized to improve the overall compression ratio on FASTQ formatted files.

## Background

DNA sequencing provides fundamental data for many research areas e.g. genomics, bioinformatics, and biology [1]. Rapid progress has been made for DNA sequencing technologies, especially the high-throughput next-generation sequencing (NGS), in the last few years. Newly proposed high efficiency methods significantly stimulate the production and usage of NGS data [2]. However the exponential growth of NGS data poses huge challenge to data storage and transmission [3]. Thereby efficient compression algorithms are required.

General-purpose compression algorithms e.g. gzip and bzip2 usually fail to obtain satisfactory results on NGS data, because they are designed for ordinary plain text or binary files. To achieve higher compression ratio, many specialized methods are proposed. For instance, Cox *et al*. [4] proposed a Burrws-Wheeler transform based compression algorithm for large scale DNA sequence databases. Jones *et al*. [5] presented Quip, a high efficient reference-based NGS data compression tool relying on external reference genomes or light-weight *de novo *assembly to generate reference sequences from the target data. Popitsch *et al*. [6] proposed the NGC tool for SAM format files compression. Hach *et al*. [7] proposed SCALCE by introducing locally consistent parsing in data encoding. More comprehensive review on NGS data compression can be found in [8,9].

Typically, raw NGS data includes a series of sequencing records (called reads). Each record consists of three major components: a metadata containing the read name, platform, and other useful information; a DNA sequence read obtained from one fold of the oversampling; and a quality score sequence estimating accuracies of the corresponding DNA bases. Existing algorithms usually focus on the compression of DNA sequence reads, and utilize conventional methods e.g. Huffman coding and run-length encoding (RLE) to compress quality scores [10]. Quality score data is considered more important than the metadata, and usually occupies similar or even more space than the DNA sequences. It may pose bigger challenges for compression than DNA sequence reads, due to the larger alphabet size. By introducing compressor specific for NGS quality scores, the overall compression ratio of NGS data can be further improved.

In this paper, we propose a memetic algorithm (MA) based NGS quality scores compression algorithm, namely MMQSC. The algorithm consists of three major parts: a Huffman coding based preprocessing is conducted in the first place, followed by MA based encoding codebook design. Finally, quality score data is compressed by using the codebook. MA is widely known as a synergy of population-based evolutionary algorithm and local search or individual learning procedures. MAs are capable of solving various complex optimization problems more effectively than their conventional counterparts [11]. In this work, the self-adaptive differential evolution combining with neighborhood search (SaNSDE) [12], and Davies, Swann, and Campey with Gram-Schmidt orthogonalization (DSCG) [13], or SaNSDE-DSCG for short, are introduced to MMQSC, to optimize the NGS quality scores compression codebook, with which most repetitive short score segments are identified and represented with much shorter encoding.

In conventional MAs, each individual represents a candidate solution of the entire problem, i.e., compression codebook. Its optimization is highly complex, because the codebook consists of hundreds of quality score symbols. Multimodal optimization tries to find all or most of the multiple solutions within a population in a single simulation run [14]. Based on multimodal optimization, the MMQSC uses an individual to represent only a single specific code vector, and composes codebook with the entire evolution population. Thereby computational complexity distributed to each individual's fitness evaluation is significantly reduced.

The proposed MMQSC obtains promising performance on NGS quality scores stored in the widely used FASTQ format [15]. Experimental results on five representative NGS data sets show that MMQSC obtains better compression ratio than other state-of-the-art methods. Particularly, MMQSC is a reference-free algorithm for lossless compression, yet obtains an average compression ratio of 22.82%, i.e., 1.81 bits per quality value (BPQ) on the experimental data sets.

The remainder of this paper is organized as follows: Section II describes details of the proposed MMQSC compression algorithm. Section III presents the experimental results of MMQSC on the five real-world NGS data sets. Finally, a conclusion is provided in Section IV.

## Methods

### SaNSDE and DSCG based memetic algorithm for multimodal optimization

MA is introduced whereby the concept of "meme", which was coined by Dawkins [16], is employed within an evolutionary computation framework to improve search efficiency. Typically, MA utilizes a population-based global optimization as fundamental framework, and introduces separate local searches or 'memes' embedded in each generation of the global evolution to refine the population [17]. The procedure of a canonical MA framework is illustrated in Algorithm 1 [18].

In MAs both global search and local search strategies can be selected flexibly according to the target problem. Typically, NGS data consists of thousands or even millions of read entries, wherein each of them contains hundreds of quality score symbols. Finding a codebook for compressing such data is naturally a high-dimensional optimization problem. Differential evolution (DE) [19] is capable of solving large scale problems effectively. In this paper, a high performance DE variant namely the SaNSDE is utilized as the global optimizer. Particularly, SaNSDE uses three self-adaptive mechanisms to select mutation strategies and control parameter values. By introducing neighborhood search in the optimization process, SaNSDE obtains higher performance than conventional algorithms. Moreover, the widely-used local search strategy DSCG is introduced to increase convergence speed. DSCG is a gradient-based optimizer that searches solution space in a greedy manner. Combining the exploration of SaNSDE and exploitation of DSCG, the proposed MA obtains promising performance on quality scores compression codebook design.

**Figure 1:**
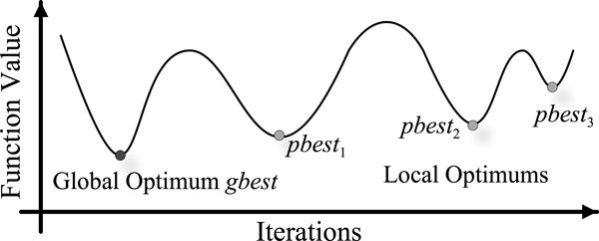
**Multimodal optimization**.

As shown in Figure 1, multimodal optimization searches for not only the global best solution *gbest *(as single-objective optimization), but also all the local optimal *pbest_i_, i *= 1, 2, 3... Multimodal optimization has been used in a wide range of applications, because it can locate all or most of the optimal solutions in a single simulation run. Fitness sharing [20] is introduced in the proposed SaNSDE-DSCG to conduct multimodal optimization. Given a raw fitness value *f_R_(**x**_i_*), wherein ***x**_i _*is the candidate solution of individual *ps_i_*. Fitness sharing transforms it into shared fitness *f_S_(**x**_i_*) using following equation:

(1) $$f_{S}\left(x_{i}\right)=f_{R}\left(x_{i}\right)\times \tau_{i}$$

in which:

(2) $$\tau_{i}=\underset{j\in \left|ps\right|,\;j\neq i}{\sum }{\left(1-\frac{d_{i,j}}{\varepsilon }\right)}^{\alpha }$$

where *ε *is the niching radius, parameter *α *controls the shape of the sharing function, distance *d_i,j _*is defined as:

(3) $$d_{i,j}=\left\{\begin{array}{c} dist\left(x_{i},x_{j}\right)\;if\;dist\left(x_{i},x_{j}\right)\leq \varepsilon \\ 0\;\;\;\;\;otherwise\;\;\; \end{array}\right)$$

where *dist(**x**_i_, **x**_j_*) is the Manhattan distance between ***x**_i _*and ***x**_j_*. If evolution population is gathering in the same optimal region, its shared fitness values will deteriorate significantly to disperse the individuals. By utilizing *f_S_(**x**_i_*) to guide the search process, SaNSDE-DSCG is capable of finding all optimums effectively.

### Compression codebook design using SaNSDE-DSCG

**Figure 2:**
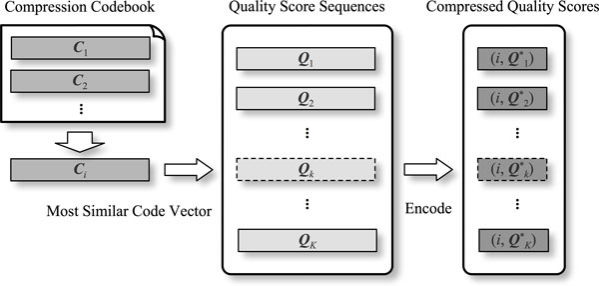
**Codebook based NGS quality score sequences compression**.

As shown in Figure 2, a NGS quality score sequence is compressed by substituting original scores with the index of its most similar code vector in the codebook, and their corresponding symbol differences.

Given a quality score sequence ***Q ***= "CCCGFF' and code vector ***C ***= "CCGHFFC". Sequence ***Q ***is encoded as {*i*, ***Q***^∗^}, where *i *is in the index of ***C***, and ***Q***^∗ ^records the symbol differences as:

(4) $$\begin{array}{c} Q=\text{C}\;\text{C}\;\;\text{C}\;\;\text{G}\;-\;\text{F}\;\text{F}\;-\\ C=\text{C}\;\text{C}\;\;\wedge \;\;\text{G}\;\text{H}\;\text{F}\;\text{F}\;\text{C}\\ Q^{*}=U\;U\;\left(I,\text{"C"}\right)\;U\;D\;U\;U\;D \end{array}$$

in which *U *denotes symbol matching (unchanged), *I *stands for insertion (marked with "∧"), *D *for deletion (marked with "−"), and *S *for substitution. For insertions and substitutions, the original symbol should also be recorded (for instance "C" on the third quality score). This matching process is conducted by using dynamic programming (DP).

Data size of the original *P*-dimensional sequence is *L_O _*= 8 × *P*, because raw quality scores are stored in 8 bits ASCII format. On the other hand, each difference type in {*U, I, D, S*} takes 2 bits to represent. Thereby the encoded data size is:

(5) $$L_{C}=\lceil log_{2}\left(M)\right\rceil +2\times P^{*}+8\times R$$

where *M *is the number of code vectors in a codebook, *P*^∗ ^denotes length of the symbol differences sequence (i.e. ***Q***^∗^), and *R *is the number of all original symbols recorded for insertions and substitutions. Given the example above, original quality score sequence takes *L_O _*= 48 bits of storage, while the encoded data uses only about 24 bits. Usually the encoding process makes *L_C _*smaller than *L_O_*, i.e., conducts compression. The more the code vector is similar to the original quality score sequence, the higher compression ratio is achieved. That is, quality of the codebook decides the overall compression performance.

In this paper, we utilize the proposed SaNSDE-DSCG to optimize compression codebook design. During the initialization, code vectors in the codebook are generated randomly, and encoded as individuals to form an evolution population. In each fitness evaluation, input candidate solution ***x**_i _*= [*x_i,1_, x_i,2_*,..., *x_i,N_*] is mapped into code vector ***C**_i _*= "*s*_1 _*s*_2 _... *s_N_*" using the following equation:

(6) $$s_{n}=S[\left\lceil x_{n}\rceil \right],n=1,2,\ldots ,N$$

where characters set ***S ***includes all unique symbols in the original quality score sequences, variable *N *is the predefined code vector length. This mapping is conducted because candidate solutions consist of continuous values, while code vectors are discrete symbol strings. The ***C**_i _*is then matched to all quality score sequences, and calculates the corresponding encoded data size. Raw fitness value of ***x**_i _*is defined as:

(7) $$f_{R}\left(x_{i}\right)=\underset{k\in K}{\sum }L_{C}\left(C_{i},Q_{k}\right)$$

in which *K *is the total number of quality score sequences, *L_C_(**C**_i_, **Q**_k_*) denotes encoded data size on the *k*th sequence ***Q**_k _*using code vector ***C**_i_*. Small *f_R_(**x**_i_*) indicates that ***C**_i _*is more similar to the original score data, i.e., obtains higher compression ratio. Shared fitness *f_S_(**x**_i_*) is then calculated accordingly.

It is noted that accurate symbol differences information, e.g. mismatched symbol positions, is not necessary for fitness values calculation. In most cases the approximate Levenshtein distance [21] is good enough to guild the codebook optimization process. Moreover, calculation of Levenshtein distance requires much less computational resources than the DP based matching algorithm. By utilizing Levenshtein distance in fitness evaluation, we can achieve similar optimization performance in a relatively high speed. Approximate size of encoded data can be calculated as:

(8) $$L_{C}^{\prime }=\lceil log_{2}\left(M)\right\rceil +2\times P_{k}+4\times lev\left(C_{i},Q_{k}\right)$$

in which *P_k _*is the length of ***Q**_k_*, and *lev*(·) denotes Levenshtein distance between the two input sequences. It's value is multiplied by 4, because there is a half chance (*I *and *S*) in {*U, I, D, S*} needs to record the original quality score. Accurate matching information is obtained only after the codebook design process for actual sequences compression.

**Figure 3:**
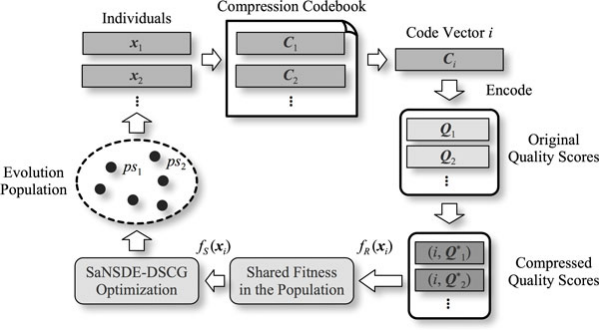
**Compression codebook design using SaNSDE-DSCG based multimodal optimization**.

Procedure of the SaNSDE-DSCG based compression codebook design algorithm is illustrated in Figure 3. In conventional single-objective optimization based design algorithms, the entire compression codebook is encoded in each individual in the evolution population. Typically, an individual in such methods is constructed by connecting all code vectors in the codebook from end to end, i.e. ***x**_i_*^' ^= {***C***_1_, ***C***_2_,..., ***C**_M_*} [22]. Optimal codebook is obtained by searching the global best solution. Dimensionality of solution space is *M *× *N*, and the algorithm calculates encoded data size (Equ. (8)) for *M *× *N *× |*ps*| time in each generation of the evolution optimization. Its computational complexity is too high to be applied on large NGS data. In contrary, MMQSC searches the solution space by using multimodal optimization, wherein each individual is utilized to represent one specific code vector, and the entire evolution population is utilized to compose the optimal compression codebook. MMQSC evolves each individual to make the code vector more representative to original quality score sequences, and accordingly the optimal codebook as a whole can compress input data more effectively. Moreover, dimensionality of solution space in MMQSC is reduced to *N*, because individual ***x**_i _*and corresponding code vector ***C**_i _*have the same length. In each generation of the evolution optimization, the encoded data size is calculated for only *M *× *N *times.

### The MMQSC algorithm

The proposed MMQSC algorithm consists of three major parts: Huffman coding based preprocessing, SaNSDE-DSCG based codebook design, and quality score data compression. Details of SaNSDE-DSCG optimization algorithm and its application on compression codebook design have been discussed in the previous sections.

During the preprocessing, raw quality score sequences are extracted from target FASTQ file, and undergo a Huffman coding. Each sequence is then converted into a symbol string by mapping every 6 bits of the encoded binary data to readable ASCII character:

(9) $$h_{t}=chr\left(33+int\left(huff\left(Q_{k}\right)\left[t\times 6\sim \left(t+1\right)\times 6\right]\right)\right)$$

in which *h_t _*is the *t*th symbol in converted string *H_k _*= "*h*_1 _*h*_2 _... *h_T _*", function *huff*(·) denotes Huffman coding, *int*(·) converts binary data to corresponding integer value, and *chr*(·) maps integer number into ASCII character. Offset value 33 is added to the input number in *chr*(·) to ensure exported character is readable. In the majority of cases *H_k _*consists of fewer symbols than original sequence ***Q**_k_*. Thereby dimensionality of code vectors is reduced, and the codebook design problem is simplified.

The SaNSDE-DSCG is conducted afterward on the encoded sequences. After optimization, MMQSC maps individuals in the evolution population into code vectors using Equ. (6) to construct a compression codebook. This codebook is then utilized to compress the input data, wherein accurate symbol differences information is obtained by using DP based matching algorithm.

Procedure of the MMQSC algorithm is demonstrated in Algorithm 2. It is worth noting that the codebook design process can also be conducted in an offline manner. That is, a universal compression codebook obtained from representative NGS quality score data sets is utilized to encode all input sequences. The time-consuming optimization process is performed for only one time. Successive compressions, which are usually conducted repeatedly, require much less computational time.

## Results and discussion

Five representative NGS data obtained from various species, and also of different read numbers and file sizes, are selected to evaluate the overall performance of MMQSC. Details of the data sets are summarized in Table 1. All data are downloaded in FASTQ format from the National Center for Biotechnology Information - Sequence Read Archive (NCBI-SRA) database [23].

**Table 1:** NGS data sets for MMQSC performance evaluation.

Data	Species	Number of Reads	Number of Bases	File Size (MB)
SRR027474	Marine metagenome	28,109	3,580,544	9.2
SRR396942	Homo sapiens	1,199,786	250,755,274	602
SRR824063	Caenorhabditis elegans	711,156	142,231,200	348
SRR824065	Caenorhabditis elegans	64,492	12,898,400	32
SRR932018	Clostridium symbiosum	169,457	8,472,850	27

In SaNSDE-DSCG optimization, the compression codebook size *M *is used as the number of individuals, i.e., |*ps*|. The value is decided as:

(10) $$M={\left(log_{2}\left(\left\lceil \frac{K}{10}\right\rceil \right)\right)}^{2}$$

The length of code vectors, i.e. dimensionality of solution space, is calculated using the following equation:

(11) $$N=\frac{1}{2}\times \left({\text{min}}_{k\in K}P_{k}+{\text{max}}_{k\in K}P_{k}\right)$$

Parameters setting for SaNSDE-DSCG based multimodal optimization is listed in Table 2 in which |***S***| is the number of unique symbols in the original quality scores, and FEs denotes the maximum fitness evaluation calls of the optimization.

**Table 2:** Parameters setting for SaNSDE-DSCG optimization.

Parameter	Population Size	Dimension	Range	*ε*	*α*	FEs
**Value**	*M*	*N*	(0, |***S***|)	0.1 × *N*	50	1E+4

Five widely used compression algorithms including the RLE, Huffman coding, gzip, bzip2, and Lempel-Ziv-Markov chain algorithm (LZMA) are utilized for comparison with the proposed algorithm. All algorithms are compared in terms of compression ratios (CR) and bits per quality value (BPQ). The BPQ is defined as follows:

(12) $$BPQ=\frac{{\sum_{k\in K}\text{min}}_{i\in M}L_{C}\left(C_{i},Q_{k}\right)}{\sum_{k\in K}P_{k}}$$

Compression results of all algorithms on the NGS data sets are summarized in Table 3.

**Table 3:** Compression performance on experimental NGS data sets.

		SRR027474	SRR396942	SRR824063	SRR824065	SRR932018
RLE	CR (%)	38.95	60.52	54.97	47.27	64.29
	BPQ	3.11	4.84	4.40	3.78	5.14

Huffman	CR (%)	53.22	60.83	42.35	58.12	49.30
	BPQ	4.26	4.87	3.39	4.65	3.94

gzip	CR (%)	22.70	35.94	30.33	26.79	30.25
	BPQ	1.82	2.88	2.43	2.14	2.42

bzip2	CR (%)	16.23	31.12	25.84	**21.14**	25.07
	BPQ	1.30	2.49	2.07	**1.69**	2.01

LZMA	CR (%)	17.63	31.32	25.63	23.08	25.00
	BPQ	1.41	2.51	2.05	1.85	2.00

MMQSC	CR (%)	**14.38**	**30.96**	**18.63**	27.38	**22.75**
	BPQ	**1.15**	**2.40**	**1.49**	2.19	**1.82**

Results in Table 3 show that, the proposed MMQSC obtains better performance than the counterpart representative algorithms. Particularly, MMQSC obtains average compression ratio of 22.82%, resulting in an over 77.18% size reduction in the quality score data. The average 1.81 BPQ result is much smaller than the original 8 BPQ in ASCII format. Moreover, performance of MMQSC remains stable in the experimental data sets, indicating that the algorithm has good robustness on different types of NGS data.

**Figure 4:**
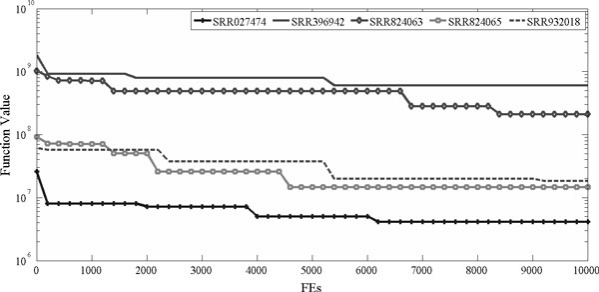
**Convergence trace of compression codebook optimization on experimental data sets**.

Convergence trace of codebook optimization processes on experimental data sets is illustrated in Figure 4, in which y-axis, labeled as function value, is the optimal shared fitness value in SaNSDE-DSCG optimization. The figure shows that by combining SaNSDE and DSCG in an MA framework to conduct multimodal search, compression codebook is optimized effectively. Particularly, DSCG increases convergence speed in the early stage of optimization. Premature convergence is successfully prevented by using the high performance SaNSDE algorithm.

## Conclusions

This paper presents MMQSC, a MA based NGS quality scores compression algorithm. The MMQSC utilizes Huffman coding to preprocess raw quality score sequences stored in FASTQ format. To obtain higher performance, a SaNSDE and DSCG based MA is proposed to optimize the compression codebook design. The Levenshtein distance is used in fitness evaluations to estimate encoded data size, and improves computation speed. After the codebook optimization, a DP based matching algorithm is conducted to obtain accurate symbol differences information. This information, as well as the optimized codebook, is utilized to compress input quality score data. Experimental results on five NGS data show that the proposed MMQSC obtains higher compression ratio than counterpart state-of-the-art algorithms. Particularly, MMQSC reduces about 77% of the storage space on the experimental data sets.

## Competing interests

The authors declare that they have no competing interests.

## Authors' contributions

JRZ and ZJ conceived and designed the study. JRZ, ZXZ, and SH performed the experiments. JRZ and ZXZ wrote the paper. ZJ, ZXZ, and SH reviewed and revised the manuscript. All authors read and approved the manuscript.

Algorithm 1 - Canonical memetic algorithm framework

1:  **BEGIN**

2:  **Initialize: **Randomly generate an initial population |*ps*|;

3:  **while **stopping criterion is not satisfied **do**

4:      Evolve population |*ps*| using global optimization;

5:      **for **each individual *ps_i _*in |*ps*| **do**

6:          Improve *ps_i _*using local searches;

7:      **end for**

8:  **end while**

9:  **END**

Algorithm 2 - Procedure of MMQSC algorithm

1:  **BEGIN**

2:  ***Preprocessing*:**

3:  Obtain raw quality score sequences {***Q***_1_, ***Q***_2_,..., ***Q**_K_*} from target FASTQ file;

4:  Conduct Huffman coding, convert input sequences into {***H***_1_, ***H***_2_,..., ***H**_K_*};

5:  ***Compression Codebook Design*:**

6:  Randomly generate evolution population |*ps*|;

7:  **while **stopping criterion is not satisfied **do**

8:      Evolve population |*ps*| using SaNSDE;

9:      Calculate raw fitness values for |*ps*| using Equ. (7);

10:      Calculate shared fitness values using Equ. (1);

11:      **for **each individual ***x**_i _*in |*ps*| **do**

12:          Improve ***x**_i _*using DSCG;

13:          Calculate raw fitness value *f_R _(**x**_i_*) using Equ. (7);

14:          Calculate shared fitness value *f_S _(**x**_i_*) using Equ. (1);

15:      **end for**

16:  **end while**

17:  Construct optimal compression codebook using |*ps*|;

18:  ***Quality Scores Compression:***

19:  Obtain accurate symbol differences information using DP based matching;

20:  Encode each quality score sequence ***Q**_k _*with corresponding code vector index and symbol differences information as (*i, **Q**_k_*^∗^);

21:  **END**

## Declarations

Publication charges for this article have been funded by the National Natural Science Foundation of China (61171125).

This article has been published as part of *BMC Bioinformatics *Volume 15 Supplement 15, 2014: Proceedings of the 2013 International Conference on Intelligent Computing (ICIC 2013). The full contents of the supplement are available online at http://www.biomedcentral.com/bmcbioinformatics/supplements/15/S15.

## References

[B1] You ZH Yin Z Han K Huang DS Zhou XB A semi-supervised learning approach to predict synthetic genetic interactions by combining functional and topological properties of functional gene network BMC Bioinformatics 2010 11 343 20573270 10.1186/1471-2105-11-343 PMC2909217

[B2] Bonfield JK Mahoney MV Compression of FASTQ and SAM format sequencing data PloS One 2013 8 59190 10.1371/journal.pone.0059190 PMC3606433 23533605

[B3] Li H Homer N A survey of sequence alignment algorithms for next-generation sequencing Briefings in Bioinformatics 2010 11 473 483 20460430 10.1093/bib/bbq015 PMC2943993

[B4] Cox AJ Bauer MJ Jakobi T Rosone G Large-scale compression of genomic sequence databases with the Burrows-Wheeler transform Bioinformatics 2012 28 1415 1419 22556365 10.1093/bioinformatics/bts173

[B5] Jones DC Ruzzo WL Peng X Katze MG Compression of next-generation sequencing reads aided by highly efficient *de novo *assembly Nucleic Acids Research 2012 40 171 10.1093/nar/gks754 PMC3526293 22904078

[B6] Popitsch N von Haeseler A NGC: lossless and lossy compression of aligned high-throughput sequencing data Nucleic Acids Research 2013 41 27 10.1093/nar/gks939 PMC3592443 23066097

[B7] Hach F Numanagic I Alkan C Sahinalp SC SCALCE: boosting sequence compression algorithms using locally consistent encoding Bioinformatics 2012 28 3051 3057 23047557 10.1093/bioinformatics/bts593 PMC3509486

[B8] Zhu Z Zhang Y Ji Z He S Yang X High-throughput DNA sequence data compression Briefings in Bioinformatics 2013 bbt087 10.1093/bib/bbt087 24300111

[B9] Giancarlo R Rombo SE Utro F Compressive biological sequence analysis and archival in the era of high-throughput sequencing technologies Briefings in Bioinformatics 2013 bbt088 10.1093/bib/bbt088 24347576

[B10] Janin L Rosone G Cox AJ Adaptive reference-free compression of sequence quality scores arXiv Preprint 2013 arXiv:1305.0159 10.1093/bioinformatics/btt257 23661694

[B11] Moscato P Cotta C Mendes A Memetic algorithms New Optimization Techniques in Engineering 2004 New York: Springer 53 85

[B12] Yang Z Tang K Yao X Self-adaptive differential evolution with neighborhood search Proceedings of IEEE Congress on Evolutionary Computation: 1-6 June 2008 2008 Hong Kong 1110 1116

[B13] Nguyen QH Ong YS Lim MH A probabilistic memetic framework IEEE Transactions on Evolutionary Computation 2009 13 604 623

[B14] Singh G Deb K Comparison of multi-modal optimization algorithms based on evolutionary algorithms Proceedings of Genetic and Evolutionary Computation Conference: 8-12 July 2006 2006 Seattle 1305 1312

[B15] Cock PJ Fields CJ Goto N Heuer ML Rice PM The Sanger FASTQ file format for sequences with quality scores, and the Solexa/Illumina FASTQ variants Nucleic Acids Research 2010 38 1767 1771 20015970 10.1093/nar/gkp1137 PMC2847217

[B16] Dawkins R The Selfish Gene 2006 UK: Oxford University Press

[B17] Huang DS Du JX A constructive hybrid structure optimization methodology for radial basis probabilistic neural networks IEEE Transactions on Neural Networks 2008 19 2099 2115 19054734 10.1109/TNN.2008.2004370

[B18] Chen X Ong YS Lim MH Tan KC A multi-facet survey on memetic computation IEEE Transactions on Evolutionary Computation 2011 15 591 607

[B19] Storn R Price K Differential evolution-a simple and efficient heuristic for global optimization over continuous spaces Journal of Global Optimization 1997 11 341 359

[B20] Sareni B Krahenbuhl L Fitness sharing and niching methods revisited IEEE Transactions on Evolutionary Computation 1998 2 97 106

[B21] Gooskens C Heeringa W Perceptive evaluation of Levenshtein dialect distance measurements using Norwegian dialect data Language Variation and Change 2004 16 189 207

[B22] Huang DS Radial basis probabilistic neural networks: model and application International Journal of Pattern Recognition and Artificial Intelligence 1999 13 1083 1101

[B23] Leinonen R Sugawara H Shumway M The sequence read archive Nucleic Acids Research 2011 39 suppl 1 19 21 21062823 10.1093/nar/gkq1019 PMC3013647

